# Methodological Challenges in Web-Based Trials: Update and Insights From the Relatives Education and Coping Toolkit Trial

**DOI:** 10.2196/15878

**Published:** 2020-07-17

**Authors:** Heather Robinson, Duncan Appelbe, Susanna Dodd, Susan Flowers, Sonia Johnson, Steven H Jones, Céu Mateus, Barbara Mezes, Elizabeth Murray, Naomi Rainford, Anna Rosala-Hallas, Andrew Walker, Paula Williamson, Fiona Lobban

**Affiliations:** 1 Division of Health Research Spectrum Centre for Mental Health Research Lancaster University Lancaster United Kingdom; 2 Department of Biostatistics Clinical Trials Research Centre University of Liverpool Liverpool United Kingdom; 3 Division of Psychiatry University College London London United Kingdom; 4 Division of Health Research Lancaster University Lancaster United Kingdom; 5 Research Department of Primary Care and Population Health University College London London United Kingdom

**Keywords:** randomized controlled trial, research design, methods, internet, web, mental health, relatives, carers

## Abstract

There has been a growth in the number of web-based trials of web-based interventions, adding to an increasing evidence base for their feasibility and effectiveness. However, there are challenges associated with such trials, which researchers must address. This discussion paper follows the structure of the Down Your Drink trial methodology paper, providing an update from the literature for each key trial parameter (recruitment, registration eligibility checks, consent and participant withdrawal, randomization, engagement with a web-based intervention, retention, data quality and analysis, spamming, cybersquatting, patient and public involvement, and risk management and adverse events), along with our own recommendations based on designing the Relatives Education and Coping Toolkit randomized controlled trial for relatives of people with psychosis or bipolar disorder. The key recommendations outlined here are relevant for future web-based and hybrid trials and studies using iterative development and test models such as the Accelerated Creation-to-Sustainment model, both within general health research and specifically within mental health research for relatives. Researchers should continue to share lessons learned from conducting web-based trials of web-based interventions to benefit future studies.

**International Registered Report Identifier (IRRID):**

RR2-10.1136/bmjopen-2017-016965

## Introduction

### Background

There has been rapid growth in the development of digital interventions [1]. One of the many challenges for researchers is testing the effectiveness of web-based interventions, whether through a web-based or hybrid (web-based and offline) trial, or a more iterative process such as the Accelerated Creation-to-Sustainment (ACTS) model [2], to meet the National Institute for Health and Care Excellence (NICE) standards for evidence-based web-based interventions. Other challenges include developing digital intervention theories, methods for detailed economic evaluation, assessing the generalizability of results to determine what works for whom in what context [3], and finding systems and approaches that can be easily adopted by the UK National Health Service (NHS) or other health care providers.

Murray et al [4] explored the methodological challenges associated with web-based trials of web-based interventions (recruitment, randomization, fidelity of the intervention, retention, and data quality), drawing upon learning from the Down Your Drink (DYD) web-based trial. The main challenges they faced were the risk of participants undermining randomization by reregistering with different details, difficulties in collecting any objectively measured data, and the high rate of attrition.

Since then, there have been a number of reviews of the methodological challenges associated with evaluating digital interventions [5-7], and many studies have focused on specific challenges such as recruitment [8-11], retention [12-19], and intervention engagement [17,18,20-26].

This paper updates Murray et al’s [4] analysis by sharing learning from a national web-based randomized controlled trial (RCT) of an intervention aimed at reducing distress in relatives of people with psychosis or bipolar disorder (Relatives Education and Coping Toolkit [REACT]; Textbox 1). The methods and results of the REACT trial are presented elsewhere [27,28]. The focus on relatives is novel and timely given the growing recognition that relatives of people with severe mental health problems provide a large amount of vital unpaid care [29] at huge personal cost [30,31], yet lack the information and support they need [32-34]. A review of the quality of mental health services has identified improving support for relatives as a national priority [35], and evidence suggests that this could be done effectively through web-based interventions [36,37]. Indeed, relatives may be a particularly appropriate population for digital interventions as they may not be available for face-to-face contact, given their caregiving commitments. Sharing learning on the most successful methods to evaluate web-based interventions within this population is therefore valuable. To date, there are no published papers dealing with the methodological challenges associated with web-based trials for relatives of people with severe mental health problems. The methodological challenges and solutions outlined here are relevant for future web-based and hybrid trials and ACTS studies, both within general health research and specifically within mental health research for relatives.

**Case study: Relatives Education and Coping Toolkit randomized controlled trial.**

- *Aim*: To evaluate the clinical- and cost-effectiveness of a web-based, peer-supported–self-management intervention, the Relatives Education and Coping Toolkit (REACT), for relatives of people with psychosis or bipolar disorder.
- *Methods*:
  - Design: A primarily web-based, two-arm, pragmatic, observer-blind, randomized controlled superiority trial.
  - Setting: The World Wide Web.
  - Participants: Based in the United Kingdom, English speaking, currently distressed, and help-seeking relatives of people with psychosis or bipolar disorder, aged ≥16 years, with access to the internet.
  - Intervention: The REACT toolkit providing National Institute for Health and Care Excellence recommended information and support through digital, peer-supported, self-management for relatives. REACT included 12 psychoeducation modules, a peer-supported group forum, private messaging to a trained relative (REACT supporter), and a resource directory (RD), ie, a comprehensive list of existing support for relatives. Relatives also received treatment as usual (TAU).
  - Comparator: RD only, plus TAU.
  - Primary outcome: Relatives’ distress at 24 weeks, measured by the General Health Questionnaire-28 [38].
  - Procedures: Recruitment took place through mental health services in the United Kingdom, charities, media, social media, and web-based advertisements. Consent, baseline data collection, and randomization were undertaken on the web through a secure system hosted at Liverpool Clinical Trials Research Centre. Follow-up was conducted primarily using web-based reminder emails, supplemented by additional offline phone calls, texts, and posts. Participants received £10 (US $1.24) on follow-up completion at 12 weeks, and either £10 (US $1.24) or £20 (US $2.48), upfront or on follow-up completion at 24 weeks.

### Design

This study consists of a discussion paper based on a case study of a web-based RCT of a web-based intervention aimed at relatives of people with psychosis or bipolar disorder (Textbox 1). We have followed the structure of the original Murray et al paper [4] for each key trial parameter and present a brief update from the literature (called *past work*), including recommendations from the Murray paper. This literature update is based on a nonsystematic scoping search of the literature since the Murray paper (using the search term *online randomized controlled trial*). The literature update is followed by discussing our own experiences, using the REACT trial approach and results (*REACT*). Finally, we provide implications for future trials with regard to each key trial parameter (*future work*).

## Updates and Insights

### Recruitment

#### Past Work

Murray et al [4] recommend having a well-planned recruitment strategy which is piloted, advertised on well-known sites, includes patient and public involvement (PPI) input on the recruitment material, and ensures a balance between the ease of recruitment material and sufficient hurdles to ensure participants are aware of what they are agreeing to, for example, follow-ups.

Study promotion and recruitment approaches have been identified as important determinants of successful recruitment [39]. A combination of web-based and offline strategies has worked well to recruit participants into web-based trials in health research [19,40]. Despite some challenges, such as cost, the potential for misrepresentation, and potentially low recruitment rates [11], Facebook has proved to be a highly successful web-based recruitment strategy [19,41].

Previous studies have found a difference in demographics depending on whether participants were recruited on the web or offline [19,38,39], but not all studies have identified the same differences. There is some agreement that more females than males come into research through web-based strategies, such as Facebook [19,42], and evidence that more highly educated participants are more likely to be recruited offline [19]. Some studies have found that older participants are more likely to be recruited through web-based strategies [19,40], whereas others have found that younger people are more likely to be recruited on the web [11,39]. Differences in the demographic characteristics of participants by recruitment strategy suggest that increasing the breadth of recruitment sources may increase sample diversity and, therefore, the generalizability of trial findings. Continually reviewing the success of recruitment strategies during a trial may increase the likelihood of meeting recruitment targets [19].

#### Relatives Education and Coping Toolkit

In line with Murray et al’s [4] recommendations, the REACT trial used a well-planned recruitment strategy combining web-based (Twitter, Facebook, online forums, Google Ads, web-based newspaper article, and NHS and mental health charity websites) and offline (mental health teams, general practitioners (GPs), universities, radio, NHS, and service user and caregiver support groups) recruitment strategies between April 22, 2016, and September 30, 2017. Strategies and materials were coproduced with REACT supporters (trained relatives employed on the trial team), who also supported recruitment. Participants were asked how they had heard about REACT (using a drop-down menu) as part of the trial registration.

The recruitment target of 666 relatives was exceeded within the recruitment period (N=800). Participants were typically middle-aged (40-60 years: 422/800, 52.8%), white British (727/800, 90.9%), female (648/800, 81.0%), and educated to university graduate level (437/800, 54.6%). Similar demographic characteristics have been found in web-based trials of web-based smoking cessation interventions [9,19]. The highest proportion of participants were mothers (387/800, 48.3%), supporting a young adult aged ≤35 years (485/800, 60.6%) with more than half of these supporting someone with bipolar disorder (462/800, 57.8%). Most were supporting only 1 person with a mental health problem, but some (209/800, 26.1%) reported supporting two or more people, and many (457/800, 57.1%) had other dependents. Over half (485/800, 60.6%) were married or in a civil partnership. Most relatives were working: some were in full-time work (301/800, 37.6%), some were in part-time employment (188/800, 23.5%), whereas others undertook voluntary work (23/800, 2.9%). A small group (66/800, 8.3%) were unable to work because of their caregiving commitments. The vast majority had good quality home internet access (795/800, 99.4%). The population recruited was similar to that recruited in face-to-face trials of caregiver interventions [43]; therefore, although the sample was biased in terms of demographics (eg, most participants being white British, female, and mothers), there is no clear evidence that this bias was related to the trial being based on the web.

As part of a post hoc analysis, we explored the relationship between key demographic variables and web-based versus offline recruitment. There was no pattern regarding who came into the trial through web-based versus offline strategies in terms of age (N=800; χ^2^_5_=5.5; *P*=.36) or education level (N=800; χ^2^_2_=1.9; *P*=.40). However, in line with previous research [19,42], more females were recruited through web-based strategies compared with males (N=800; χ^2^_1_=16.7; *P*<.001).

The success of each recruitment strategy used was indicated by the number of randomized participants who were recruited via each strategy (Table 1). Of the randomized participants, more than half (421/800, 52.6%) were recruited through 5 primary web-based strategies, and less than half (379/800, 47.4%) were recruited through 10 primary offline strategies. The most successful strategies (Facebook and mental health teams or other professionals) were also two of the most expensive. The costs associated with Facebook related to outsourcing Facebook advertisements to an information technology company for development and maintenance. Over 13 months, 873,096 individuals were reached; 53,216 people engaged with an advertisement (liking, commenting, or sharing), and there were 71,026 clicks on the REACT website. The costs associated with mental health teams/professionals were related to service support costs for staff time, trial manager and REACT supporter time, and promotional material. We decided to use the available data on costs incurred, in line with our in-trial analytical approach, rather than hypothetical data on costs of an NHS model of referral to the intervention. Recruitment costs for the trial were calculated over the total trial period and divided by 3287, the number of people who completed eligibility screening for the trial. Cost included an average per participant in both arms of the trial, as both required recruitments. Recruitment costs for each strategy were divided by the number of participants who were recruited (and randomized) via that strategy. The components and total cost of recruitment are outlined in Table 2.

**Table 1:** Recruitment strategies for randomized participants.

Recruitment strategies for randomized participants	Web-based/offline	Values, n (%)	Approximate cost per participant (£)
Facebook	Web-based	206 (25.8)	93 (US $115.39)
Mental health teams/professionals	Offline	151 (18.9)	107 (US $132.77)
Internet search	Web-based	121 (15.1)	28 (US $34.74)
Mental health charities^a^	Web-based	77 (9.6)	38 (US $47.15)
Recommended by a friend/family	Offline	74 (9.3)	0
GP^b^	Offline	59 (7.4)	170 (US $210.94)
Caregiver or service-user support group	Offline	42 (5.3)	4 (US $4.96)
NHS^c^ contacts	Offline	25 (3.1)	—^d^
Twitter	Web-based	15 (1.9)	2 (US $2.48)
Employer	Offline	8 (1.0)	32 (US $412.51)
Other third sector organization	Offline	8 (1.0)	—
Not classifiable	Offline	6 (0.8)	—
Other public adverts (excluding NHS adverts)	Offline	4 (0.5)	—
Local newspaper	Web-based	2 (0.3)	65.4 (US $81.15)
Research team	Offline	2 (0.3)	—

^a^Mental health charities used a combination of web-based and offline strategies; however, most were web-based, and by far, the biggest recruiter was Bipolar UK, and their main means of recruitment were email-newsletters.

^b^GP: general practitioner.

^c^NHS: National Health Service.

^d^Value unknown.

**Table 2:** Recruitment costs.

Recruitment strategy	Cost (£)
Advertisements (Facebook, Google, and Bipolar UK)	11,059.56 (US $13722.70)
Printing	1526.00(US $652.66)
Flyers and postage	50.00 (US $62.04)
Total recruitment costs	12,635.56 (US $15678.20)

Despite designing a broad (throughout the United Kingdom) recruitment strategy incorporating a wide range of both web-based and offline methods, our sample predominantly comprised middle-aged, highly educated, white British females, who cared for an individual with bipolar disorder. It is possible that the predominance of highly educated individuals reflects the interest of this group in research and self-education. Moreover, the predominance of female participants could partially reflect the continuing burden of care falling to women. The lack of relatives from ethnic minority groups could result from both bias in the referral process of offline recruitment strategies and from lack of cultural adaptation in content and language, both of which were beyond the scope of this study, but is also a persistent failure across mental health services more widely.

#### Future Work

A combination of web-based and offline strategies worked well to recruit relatives of people with psychosis/bipolar disorder. Although mental health teams/professionals proved a successful recruitment avenue, recruitment targets would not have been met if this had been the only strategy used. NHS trusts and GP practices rarely have an accurate, up-to-date method of identifying relatives. Therefore, recruitment is often through the patient, who may forget or not wish to pass the information on. Bolstering offline NHS recruitment with web-based strategies such as social media is effective, with Facebook proving most successful. That said, expert knowledge of how to target Facebook adverts and test the effectiveness of different ones may be required for cost-effective advertisement.

Monitoring of recruitment strategy success is important to adapt and refocus time, money, and effort. For example, we stopped relatively unsuccessful and costly Google Ads (classified under *Advertisements* in Table 1) and concentrated on successful (yet still costly) Facebook advertisements.

Despite the extensive recruitment strategy applied in the REACT trial, predominantly highly educated, white British female participants, who cared for an individual with bipolar disorder were recruited, which creates a problem with generalizability. NHS research and development departments need to ensure better availability of caregiver data so that offline recruitment methods can be made more effective, and clinical trials (both offline and web-based) can be made more accessible. Moreover, cultural adaptation should be a key focus of future work in this area to ensure that digital health interventions do not exacerbate existing inequalities in access to health care.

### Registration Eligibility Checks

#### Past Work

Murray et al [4] did not specifically review registration and eligibility checks. Buis et al [8] analyzed *help tickets* (provided by 38% of participants) logged in a database during enrolment into a web-based RCT of a walking program and found that the most common issue was related to the study process. Being older, female, and having a lower self-rated internet ability increased the likelihood of reporting an issue during enrolment.

As with all trials, randomized participants must meet strict eligibility criteria to adhere to the protocol. In web-based trials, there is an increased risk of not detecting participants who may have misrepresented their eligibility to take part, perhaps because of seeking payment or potential access to treatment [10]. Kramer at al [10] suggested procedural (eg, not advertising payment), technical (eg, tracking internet protocol [IP] addresses to identify multiple registrations), and data analytic strategies (eg, sensitivity analyses) to address sample validity in studies using the internet for recruitment.

#### Relatives Education and Coping Toolkit

The registration process aimed to check eligibility (using web-based checkboxes), ensure consent was fully informed, avoid participants registering more than once, and encourage completion of baseline questionnaires before randomization. On the basis of PPI feedback, the landing page had limited text, included videos, and highlighted the required commitment to follow-ups (as recommended by Murray et al [4]). Strategies were also employed beyond the landing page to encourage trial registration—the inclusion of lay language to explain processes and encourage continuation, a progress bar, phone number activation (participants verified their phone number by inputting a code sent to their mobile/landline into the registration system), automated reminder emails 24 hours after consent and 7 days after baseline questionnaires were started, and the option to have direct contact with the trial manager on the web or by telephone.

Overall, 43% of those who completed the eligibility checks failed on at least one item, most commonly (81% of those failing) on the requirement to report *being strung up and nervous all the time*, *rather more than usual*, or *much more than usual* (Table 3). This item was included in the screening process to avoid a floor effect at baseline. It showed the highest item-total correlation with the total General Health Questionnaire-28 (GHQ-28) [38] scores in our feasibility study [44]. However, relatives who had been distressed for longer periods and so responded *no more than usual* were ineligible. Following contact from ineligible participants highlighting their frustration at being excluded from this item, lay language *pop-ups* were included to explain why certain criteria were important and had not been met. These *pop-ups* did not enable potential participants to change their answers, but there was nothing to stop the participant attempting to progress by reaccessing the eligibility process. This information helped participants to understand why they were not eligible and alleviated frustration (we did not receive any further contact from participants regarding issues with eligibility after the introduction of these *pop-ups*).

**Table 3:** Eligibility details.

Question	Number of participants failing eligibility for the question, n (%)
I am 16 years old or over	10 (1)
I am a relative (or close friend providing regular support) of someone with psychosis or bipolar disorder	88 (6)
Have you recently been feeling nervous and strung up all the time?	1146 (80.9)
I would like to receive help for my distress through an online toolkit	118 (8.3)
I have regular access to a computer which is connected to the internet	28 (2)
I have a good working knowledge of written and spoken English language	13 (1)
I live in the UK^a^	13 (1)
To the best of my knowledge, I am the only relative/close friend of the person I support taking part in the REACT^b^ study	67 (5)

^a^UK: United Kingdom.

^b^REACT: Relatives Education and Coping Toolkit.

#### Future Work

Our Relatives Advisory Group (RAG) recommended the following strategies for the REACT trial: limiting text, adding videos highlighting trial commitment, using lay language to explain the process, progress bars, reminder emails, and personal contact. Feedback from users of the site agreed with these recommendations. Therefore, similar strategies should be employed and could potentially encourage continued engagement with the registration process. Future trials should consider recording reasons why people ask for help during the registration process to provide personalized feedback and improve the design of the process.

Careful consideration is needed regarding eligibility questions to avoid being too inclusive or exclusive of participants. Providing lay language *pop-ups* to explain ineligibility and including suggestions for other relevant studies or sources of support is particularly recommended to reduce participant frustration.

PPI feedback is essential for all recruitment materials and for designing trial landing and registration pages.

### Consent and Participant Withdrawal

#### Past Work

Consent was not specifically reviewed in Murray et al’s paper [4]. Valid informed consent must be obtained for all trial participants in line with ethical standards, including the General Data Protection Regulation (GDPR) [45]. The British Psychological Society (BPS) [46] recommends that internet-mediated research fully informs participants regarding times at which, and ways in which they can withdraw from a study.

Despite the guidelines and recommendations on internet-mediated research, there is a general lack of guidance on assessing the capacity to consent and the practicalities of the withdrawal process for web-based trials.

In line with the Privacy and Electronic Communications Regulations (PECR) [47], Murray et al [4] suggest that all emails include information on the withdrawal process.

#### Relatives Education and Coping Toolkit

With regard to consent, the BPS and Health Research Authority (HRA) guidelines for internet-mediated research were adhered to [46,48]. These include providing checkboxes, limiting the length of the consent form, and providing participants with sufficient detail about the study, their participation, and potential risks in the participant information sheet (PIS). The PIS was available on the web and presented as part of the registration process before the web-based consent form. NHS ethical approval was obtained from the Lancaster National Research Ethics Service Committee (15/NW/0732).

As suggested by Murray et al [4], information regarding the withdrawal process was included in the PIS, and a link to allow withdrawal was contained in all follow-up reminder emails. Participants could withdraw at 3 different levels (from 12-week follow-up but not 24-week follow-up; from all follow-ups but continue use of REACT or the resource directory [RD]; or from all follow-ups and REACT or RD) and were asked to provide a reason for their withdrawal from a drop-down menu with the following options: *I don’t have time due to other commitments*; *I didn’t like the website I was given*; *I don’t like filling in the questionnaires*; *I don’t feel well enough to take part*; or *Other (please specify)*. Participants could withdraw on the web or via contact with the trial manager, who would then add the relevant information to the web-based dashboard, including the level of withdrawal and reason if given.

A total of 800 participants completed the web-based consent procedures and entered the trial. Of these, 5.8% (46/800) of participants withdrew from the trial (6 from 12-week follow-up only, 26 from all remaining follow-ups, and 14 from all remaining follow-ups and intervention). A total of 7 participants reported that their withdrawal was because of lack of time due to other commitments; 4 reported they did not like the website they were given; 4 reported they did not like filling in the questionnaires; 4 reported that they did not feel well enough to take part; and 31 gave a combination of other reasons (not categorized).

#### Future Work

Researchers should follow guidelines (eg, GDPR, BPS, and HRA) for gaining web-based consent, as outlined earlier. Despite following guidelines for internet-mediated research, the big challenge that we were unable to address was the ethical issue of assumed capacity in web-based trials. There is a lack of face-to-face contact between researchers and participants, which results in missing dialogue and interaction required to establish the capacity to consent. Future research would benefit from reviewing the current range of web-based interventions and the types of risk they present, including the assumed capacity to consent.

Participants must be given the option to withdraw from the trial at any time, and it is important for researchers, especially those conducting a web-based trial where contact is remote, to work out exactly what participants wish to withdraw from. This can be done by offering participants options to withdraw from different levels of the trial, for example, the research versus the intervention. Identifying and sharing reasons for withdrawal may help to reduce attrition in future web-based trials of web-based interventions.

### Randomization

#### Past Work

The biggest risk to the validity of the randomization process in web-based trials is the reregistration of participants who were not allocated to the trial arm they hoped for. Murray et al [4] took steps to reduce the risk of reregistration— email validation, removal of incentive to reregister, and monitoring of potential reregistrations (through offline contact details and IP addresses). Although not infallible strategies, there was no evidence to suggest that reregistration was a significant issue. Therefore, the steps implemented may have been adequate, and more draconian approaches may put people off registering at all.

#### Relatives Education and Coping Toolkit

Recommendations from the DYD trial were followed in the REACT RCT. Specifically, a validated email address and phone number (mobile or landline) were a requirement for registration and checked for prior use. The advantages of the comparator (RD) were highlighted, and it was made clear in the PIS that the RD group would be given access to the content of the REACT modules at the end of the trial; financial incentives were provided to both arms only after baseline was complete and for follow-up (further details in the section Retention); and participant contact details were verified as unique (ie, did not match any already registered) by automated univariate monitoring of data fields for email, mobile number, landline number, postcode, and address. We chose not to use IP address checks to monitor registration because of the risk of the ease with which a participant can access a website from a different IP address, or the risk that we could block participants using a shared external IP address (eg, where an organization has 1 external facing IP address but many internal addresses).

It was also made clear in the PIS that only 1 relative per service user should register for the trial because people who are caring for the same individual are likely to have related levels of distress, and that log-in details should not be shared. We could not explicitly check whether only 1 person per service user registered because we did not collect any data about the service user. However, based on the assumption that many caregivers would be immediate relatives and immediate relatives are more likely to live with the person they care for, we checked that relatives’ addresses were unique.

There was no evidence that multiple registrations were an issue, based on system checks for duplicate email addresses, phone numbers, and postal addresses.

#### Future Work

Although there was no evidence that multiple registrations were an issue in the DYD or REACT trials, this was possibly because of the steps followed to reduce this risk. Researchers should aim to validate contact details, remove incentives to reregister, and collect and monitor offline contact details as precautionary measures.

### Engagement With Web-Based Interventions

#### Past Work

To ensure fidelity of the intervention (ie, how much a participant uses the intervention and which modules/sections), a detailed description of the development of the DYD intervention was published [49], and participant use of the intervention was automatically monitored. The authors suggest that considerable preparatory work is required to understand how and why the intervention is likely to work.

Studies have suggested an association between greater engagement (higher intervention use) and improved outcomes [22,50]; however, for some, 1 visit can be enough to elicit a positive change [18]. The problem with measuring intervention use in all trials is knowing what *meaningful engagement* is—more engagement does not necessarily mean more effective engagement [26]. Understanding how and how much participants use web-based interventions is particularly challenging as participants are free to use the intervention when and how they wish (unlike if they were receiving face-to-face therapy in an offline trial).

#### Relatives Education and Coping Toolkit

To ensure that the REACT intervention was engaging for users, PPI was an important part of the development of the resource and running the REACT trial. Relatives were involved in the development of the initial content for the toolkit [51] and in subsequent iterations to develop a web-based version of REACT [52].

Moreover, we chose to employ peer workers—relatives with lived experience of supporting someone with a mental health problem—to support the website. The benefits of employing peer workers were highlighted in the design workshops that we conducted to develop the web-based version of REACT [52], and have been evidenced by research showing benefits both for those receiving support and for the peers themselves [53-56]. Furthermore, our perception was that relatives (peer workers) would be highly knowledgeable, empathetic, and motivated to support other relatives, making the intervention more engaging. Developing the peer worker/REACT supporter role as part of the NHS workforce was also consistent with recommendations from NICE in 2016.

Relatives were also involved in advisory/consultant roles to ensure that their input was recognized in the delivery and steering of the trial, including all main trial parameters. This included an advisory panel to consult with about trial processes and the content of the REACT toolkit and RD and the Trial Management Group and Trial Steering Committee (TSC), enabling them to influence key decision making relevant to the REACT trial and engaging users.

Although a description of REACT has been published [52,56], we did not specify to relatives how much or how often they should engage with REACT. Web use was measured by data showing activity on webpages (page downloads, number of log-ins, and time spent on a page), but none of these were completely accurate measures of user behavior and did not tell us anything about how this information was being processed or used. To allow for prolonged periods of inactivity when participants did not actively log off from the intervention, inactivity time on a given page was capped at 20 min. These capped values were replaced with the mean total time spent on the given page by REACT participants.

The most popular module (visited by 52% of REACT participants) was the REACT group forum, where relatives could share experiences with each other in a safe, online community facilitated by REACT supporters.

There was no statistically significant causal impact of intervention use (in terms of the number of webpage downloads [*P*=.30; *Z*=−1.1], number of log-ins [*P*=.30; *Z*=−1.0], and time spent on REACT [*P*=.30; *Z*=−1.1], assessed using instrumental variable regression) on the outcome (in terms of a reduction in distress at 24 weeks according to the GHQ-28).

#### Future Work

On the basis of our findings, the most popular section of the intervention was the interactive forum [28]. Therefore, including an interactive forum in web-based interventions may attract more participants; however, care should be taken to adequately facilitate such modules to manage risk (see section Risk Management and Adverse Events).

Although we were able to measure intervention use, we were unable to specify what an ideal level or pattern of use was likely to be. This is particularly challenging to do where the exact mechanism of action is unknown, and the intervention sites link out to other sites, for example, the RD in the REACT intervention, which appeared to be used for a very small amount of time, but may have led to participants using the information to access other relevant resources that we did not monitor. It may be informative to track user traffic to other sites in these circumstances, to understand what information and support participants are getting, and qualitatively explore how they are using this information. Patterns of use alone could helpfully be supplemented by prespecified process evaluations to understand in a deeper context, the implementation, and mechanisms of impact.

### Retention

#### Past Work

The primary method of follow-up in the DYD trial was email (an initial email followed by up to 3 reminders) containing a link to follow-up questionnaires. A subsample of nonresponders was studied at 3 months (final follow-up) to see if offline (postal or phone) reminders were helpful. Murray and colleagues found that offline follow-up was poor, possibly because of participants’ desire for anonymity, which resulted in less than one-third of participants providing offline contact details [4]. The authors suggest careful consideration of offline follow-up, given the additional expense and time needed compared with web-based follow-up.

The loss to follow-up is still one of the biggest challenges for web-based trials [6], jeopardizing statistical power, and therefore the generalizability, reliability, and validity of results [57]. Previous research suggests that being female, older, and better educated predicts greater response to follow-up [23,58], as well as being white and having good internet skills [59,60]. Reminding participants of the importance of completing follow-ups and providing them with incentives may reduce follow-up attrition [13]. Research suggests that providing a higher financial reward increases retention to follow-up [12,15,18].

#### Relatives Education and Coping Toolkit

Drawing on recommendations from the DYD trial and other previous studies [12,15,61,62], we used several strategies to maximize follow-up. First, participants were only randomized once baseline assessment measures were completed (also crucial to the integrity of the trial in general). Second, detailed explanations about why data completion at follow-up was so important were included in our recruitment materials. Third, participants in the comparator arm were informed that they would be able to access toolkit modules after the final follow-up. Finally, multiple contact details were collected at registration, which allowed the research team to send multiple reminders by different methods (email, post, SMS, and phone) and with different options for completing data (based on PPI feedback), striking a balance between cost, data, and burden on participants. Finally, participants were offered a financial incentive for baseline and follow-up. The effectiveness of £10 (US $12.41) versus £20 (US $24.82) and conditional versus unconditional financial incentives at 24 weeks was tested in a study within a trial.

The REACT RCT achieved a 72.8% (582/800) retention rate at the primary outcome point (24 weeks), surpassing our minimum threshold for adequate study power (70%) and that achieved in the DYD trial (48%). As part of a post hoc analysis, we explored the relationship between key demographic, recruitment, and intervention use variables, and retention. There was a statistically significant association between age and retention at 24 weeks follow-up (older participants were more likely to provide data at 24 weeks; N=800; χ^2^_5_ =15.31; *P*<.009), but not for gender (N=800; χ^2^_1_=3.52; *P*<.06), or education level (N=800; χ^2^_2_ =4.06; *P>*.10). There was no statistically significant relationship between whether participants had been recruited on the web or offline and retention at 24 weeks follow-up (N=800; χ^2^_1_=2.79; *P*<.10). In line with previous research [17,22], higher intervention use (in terms of time spent on the website, number of webpage downloads, and number of log-ins) was significantly associated with greater retention at 24 weeks (*P*<.001).

The highest proportion of participants completed follow-up on the web after the initial email request to do so; however, there was a cumulative effect of additional reminders (Table 4).

There was no effect of higher versus lower or conditional versus unconditional incentives on follow-up completion.

**Table 4:** Participant completion of the primary questionnaire after each reminder.

Reminder	Web-based or offline retention strategy	Participants who completed the questionnaire at 12 weeks (n=594)^a^, n (%)	Participants who completed the questionnaire at 24 weeks (n=599), n (%)
Completed on the web after first reminder email	Web-based	177^a^ (30.0)	162 (27.0)
Completed on the web after second reminder email	Web-based	114 (19.1)	71 (11.9)
Completed on the web after third reminder email	Web-based	61 (10.2)	93 (15.5)
Completed on the web after manual text message	Offline	80 (13.5)	94 (15.7)
Completed GHQ-28^b^ over the phone or on the web after phone call	Offline	68 (11.4)	81 (13.5)
Completed GHQ-28 via post or on the web after receiving a postal pack	Offline	84 (14.1)	76 (12.7)
Completed GHQ-28 via auto text	Web-based	10 (1.7)	22 (3.7)

^a^Five patients were not sent a 12-week reminder email, 2 of these were because of issues with the reminder system, and 3 patients completed the 12-week follow-up at 11 weeks postrandomization, that is, before the first reminder was sent.

^b^GHQ-28: General Health Questionnaire-28.

#### Future Work

Despite additional offline forms of contact (SMS, phone call, and postal) being time consuming in the REACT RCT, it is unlikely that people who completed these strategies would have done so otherwise. Therefore, with careful consideration, a combination of web-based and offline retention strategies is likely to be most effective for retention to follow-up.

In line with previous research [19], there was no statistically significant relationship between whether participants had been recruited on the web or offline and retention, providing support for the use of both web-based and offline recruitment strategies (see Recruitment section).

In contrast to previous research [12,15,18], we found no effect of higher versus lower and conditional versus unconditional incentives on follow-up completion, which might reflect differences in the populations and therefore highlight the importance of understanding the motivations of the population being recruited (for which PPI involvement is crucial). For example, it is possible that older relatives in a caring role in REACT are more motivated to take part in the study by having access to the intervention and the opportunity to improve care for other relatives, whereas younger people recruited to a sexual health or smoking cessation study [12,18] may have less disposable income and be more motivated by the financial reward. However, this remains speculative and is an important area for future research. Care should be taken to avoid ineffective strategies or strategies that could undermine pre-existing motivations, such as altruism.

### Data Quality and Analysis

#### Past Work

Potential bias because of constraints on how data are collected, independent verification of data, missing data, and low follow-up rates are important considerations for web-based trials [4]. In the DYD trial, data quality at baseline and follow-up was good in terms of useable data. To maximize data quality, Murray et al [4] collected baseline data before randomization, maximized the credibility of the comparator, used a primary outcome measure developed for web use, and checked reliability and validity. They also minimized the use of free text in data collection by using drop-down menus or forced choice, required participants to complete mandatory questions, did not allow participants to provide unusable data, and piloted questionnaires. The authors recommended an active PPI group to provide feedback on data quality and collaboration between statisticians and programmers to ensure data are collected and stored in a useable format.

Murray et al [4] also suggested that researchers address some of the challenges with web-based trials during analysis, for example, through intention-to-treat and sensitivity analyses.

We are not aware of any updates from the literature.

#### Relatives Education and Coping Toolkit

The REACT RCT followed the procedures and recommendations from the DYD trial, and also reviewed the response rates and completeness of the primary outcome regularly.

In the REACT trial, both intention-to-treat and sensitivity analysis were used along with mean imputation to replace capped inactivity for website use.

Intention-to-treat (ie, analysis according to the randomized group, regardless of whether the participant engaged with their randomized intervention) was the primary analysis approach. This was supplemented by causal analyses of the primary outcome, using instrumental variable regression with continuous measures of web usage (total number of webpage downloads from the REACT intervention website, total time spent logged on the REACT intervention website, and the total number of log-ins to the REACT intervention website) to assess the impact of actual intervention use on the outcome.

A joint modeling approach (using baseline, 12-week, and 24-week outcome data) was used to assess the impact of missing data at 24 weeks on the conclusions drawn from the analysis of each primary and secondary efficacy outcome. This analysis assessed whether there was any difference in outcome (here the longitudinal outcome rather than the outcome at 24 weeks alone) between the randomized arms adjusted for missingness by inherently allowing for the correlation between patterns in missingness and outcome.

Sensitivity analyses were employed to assess the impact of late data completions on the primary outcome (eg, excluding results received beyond 27 weeks for the 24-week primary outcome).

Participants scoring higher on the GHQ-28 at baseline were more likely to drop out at follow-up; therefore, data were not missing at random. Accounting for this was important as analyses appeared to show some statistically significant benefit of REACT over RD only at follow-up on how supported relatives felt, which did not remain after accounting for missing data.

Despite relatively good quality effectiveness data, some of the health economics (HE) data were poor. For example, because of the lack of quality checks, data on the use of medicines was not usable. These issues were likely because of the health economist not being involved in designing the data collection process, lack of adequate testing at the outset, some items not having mutually exclusive options in drop-down menus, and occasional failure of links to follow-on questions. Unfortunately, HE evaluation was not conducted as part of the feasibility study for REACT, which would have identified issues with the HE data earlier.

#### Future Work

Extensive testing of data collection procedures and quality is recommended. Data should be reviewed early in the trial to check whether they are being collected correctly and to check for missing data. Future web-based trials would benefit from including a health economist in designing the data collection process and, when possible, conducting an economic evaluation at the feasibility trial stage.

### Spamming

#### Past Work

For mass mailings to be legal, they must have an *unsubscribe* option [4]. One participant in the DYD trial suggested repeat follow-up reminder emails verged on being spam because of no obvious way to withdraw permission. Emails were amended to remind participants that they could withdraw at any time.

We are not aware of any updates from the literature.

#### Relatives Education and Coping Toolkit

As per PECR [47] rules and legislation around clinical trials, in the REACT trial, we included the following on all follow-up emails: “P.S. Please remember that you can choose not to complete this follow-up without giving a reason. However, if you feel you cannot (or do not want to) complete this follow-up, it would be really helpful for the future if you could tell us why. Please click here [link to withdraw included].” There was no evidence that spamming was an issue.

#### Future Work

Participants must be contacted several times during a longitudinal study such as a trial; in web-based trials of web-based interventions, this is often via email and may be a combination of reminders to complete follow-up and reminders to visit the intervention website, creating a lot of email traffic. Working closely with PPI groups and ethics committees, it is important to find a way to contact participants repeatedly, without being intrusive. Participants should be given control over the number of emails received regarding the use of the intervention, as there is likely to be individual variation in what is felt to be useful. It must be clear on all emails that participants can withdraw from the trial at any time. Making email titles factual and academic rather than *friendly*, avoiding the use of phrases such as *free online support*, and considering where the email appears to have been sent from may help to avoid emails going into spam folders.

### Cybersquatting

#### Past Work

Cyber squatters (fake domain names based on the intervention website name) may put people off further searching if they think they have come across the original website [4]. By the end of the DYD pilot study, there were at least three cyber squatters making money from advertising other websites. The authors advise other researchers to buy all related domain names before starting research.

Quick response (QR) codes have been used on offline recruitment materials to take potential participants directly to the correct trial website [63], but can only be used on mobile phones or tablets.

#### Relatives Education and Coping Toolkit

Although the REACT website address was visible on all written recruitment materials, and all participant emails contained a direct link to the website, many participants also used web search engines. This was initially a problem because many found the website from the REACT feasibility study [44], which had a similar domain name; therefore, the feasibility website was taken down. However, a more challenging issue was that during the trial, a parallel implementation study [64] was running at the same time, and also ran from Lancaster University. This study took place in 6 trusts across the United Kingdom, each with its own REACT-related domain name. This led to some confusion, which required a complex approach of redirecting participants to ensure they were trying to access the right website.

#### Future Work

In addition to checking, and potentially buying (depending on cost), related domain names before the commencement of a trial, researchers should use direct links to the trial website on all web-based recruitment material and participant emails, and avoid having multiple, similar domain names for linked studies. An alternative is a single log-in page that redirects participants to the appropriate version of the website. QR codes can be used on offline recruitment materials to take potential participants directly to the correct trial website through their mobile phones or tablets. The effectiveness of QR codes for reducing the effects of cybersquatting in trials for relatives of people with serious mental illness requires further investigation.

### Patient and Public Involvement

#### Past Work

PPI was not specifically reviewed in the paper by Murray et al [4] on the DYD trial.

Although participants appreciate the flexibility and convenience of web-based trials, the lack of connectedness and understanding can pose a challenge for the engagement of patients and the public involved in supporting the study (PPI) if this is also done on the web [65]. PPI in web-based trials may require new strategies to enhance engagement and ensure meaningful involvement.

#### Relatives Education and Coping Toolkit

To ensure engagement, relatives involved in the clinical delivery of REACT (REACT supporters and supervisors) were physically located with the research team, and consequently had high levels of face-to-face contact with other members of the team. However, the RAG, trial management meetings, TSC, and an independent data monitoring and ethics committee (IDMEC) all met on the web. Participants were located across the United Kingdom and this saved time and offered a cost-efficient approach.

However, feedback from participants suggested that the remote nature of contact in the RAG, TSC, and IDMEC had a detrimental impact on engagement for some members, particularly those with a role of PPI, who felt directly less engaged with the process or the other people involved and found it harder to input. They also found the technical aspects of web-based meetings to be challenging and less engaging.

#### Future Work

Effort is needed to understand the motivation for people to take part in advisory groups and oversight committees, particularly those contributing from a PPI perspective, and whether a web-based design will be able to meet their expectations. PPI input to support the delivery of the research may best be done using a combination of web-based and offline approaches to facilitate engagement, and training should be provided by the research team in both the technology and format of web-based meetings.

### Risk Management and Adverse Events

#### Past Work

Risk management was not specifically reviewed in Murray et al’s paper [4].

Identifying and responding to risk during a web-based trial needs careful consideration because of the remote nature of contact. Risk is one of the things that NHS trust staff are most concerned about when considering the promotion of web-based interventions to their service users [64], and awareness of how risk is managed is likely to facilitate staff engagement.

#### Relatives Education and Coping Toolkit

A comprehensive approach to risk was taken to identify and manage potential adverse events. The REACT website had several clear notifications that REACT was not monitored outside of working hours and could not offer crisis support. Clear signposting to places for support (including NHS services and charities such as Bipolar UK and Rethink) were included in places where risk could be picked up on the website. *Red flags* (low-risk adverse events) were raised by the web-based data capture system in response to answers to questionnaire items that indicated possible risk to self or to the person cared for. This automatically triggered a standardized email to participants, expressing concern, checking if the participant was okay and pointing them to appropriate support, and a notification email to the trial manager. Risk could also be identified by the REACT supporters through direct messaging or the forum, or the trial manager when contact was made for follow-up, resulting in a tailored version of the standardized email being sent. Identified risk events were recorded on a web-based dashboard.

High-risk adverse events (clear evidence of immediate and serious risk to life or to child welfare, leading to immediate contact with police or social services as appropriate) were reported to lead clinical contact, the TSC chair, sponsor, and NHS Research Ethics Committee. Clinical contacts were available within the team for advice on managing risk, and the trial manager and supporters were trained in risk assessment and protocols to respond to this. The supporters also received regular supervision with a clinical psychologist and peer support with each other.

Over the course of the trial, 363 participants were sent low-risk standardized automated emails (185 participants were sent more than one email), 3 low-risk adverse events in the RD were identified by the trial manager, 16 low-risk adverse events (13 from intervention and 3 from RD) were identified by the REACT supporters, and no high-risk adverse events were reported.

#### Future Work

All staff should be trained in risk management specific to the population they work with, and clear protocols provided. Managing risk on the web is likely to be a new skill for staff, and study-specific protocols are likely to be required, as clinical services may not have appropriate governance in place. It is also important that participants understand how risk will be managed to ensure they feel safe and supported.

## Key Recommendations for Designing Web-Based Trials

The key recommendations are as follows:

1. PPI in the design and delivery of all stages of the study is crucial and may be best performed using a combination of web-based and offline approaches to maximize engagement. Ensure PPI input includes cultural diversity, and that this informs recruitment and retention strategies that increase equality in access for any ethnic or demographic populations.
2. Ensure participants are directed to the right website by buying all related domain names and directing them, and considering the use of QR codes.
3. Combine web-based and offline recruitment strategies and monitor the success of each recruitment strategy during the study to inform flexible adaptation throughout recruitment.
4. Understand the motivation of participants for taking part in the trial and target incentives accordingly. Greater financial incentives may not always improve retention and could undermine altruistic motivations.
5. Where exclusion criteria are applied, explain clearly why these are necessary and signpost people who are not eligible to take part in alternative sources of help where possible to reduce frustration.
6. Reduce the risk of reregistration by those who do not meet the inclusion criteria or are not allocated their preferred intervention by requesting web-based and offline details and checking personal details for before use, offering access to both interventions at the end of the study, and ensuring financial reward is equal in both study groups and is given after baseline measures are completed.
7. Ensure all design complies with ethical and data protection guidelines, including clear instructions on how to withdraw from any elements of the process in all correspondence.
8. Carefully test data collection procedures and data quality before trial launch and throughout trial implementation. Maximize data quality by ensuring data capture is designed to: only allow valid responses, using forced-choice drop-down options where possible and systems are fully tested at the outset and regular points throughout the study.
9. Consider giving expectations for levels of use to participants and/or including a peer forum to increase levels of engagement.
10. Maximize retention by highlighting why retention is so important in both study groups, incentivizing each data collection point, using a combination of web-based and offline follow-up strategies, and offering all participants access to both interventions at the end of the study.
11. Where relevant, ensure that clear risk protocols are in place, and staff are adequately trained.

### Conclusions

Web-based trials are increasingly being used to test web-based interventions. Researchers are sharing lessons learned from conducting such trials, which is of great benefit to future studies. This paper has provided recommendations based on key considerations for web-based trials of web-based interventions (recruitment, registration eligibility checks, consent and participant withdrawal, randomization, engagement with a web-based intervention, retention, data quality and analysis, spamming, cybersquatting, PPI, and risk management and adverse events) adding to this growing literature base. The development considerations and solutions outlined here are relevant for future web-based and hybrid trials and ACTS studies, both within general health research and specifically within mental health research for relatives. The structure of what we did is also relevant for future face-to-face trials.

## Abbreviations

ACTS: Accelerated Creation-to-Sustainment
BPS: British Psychological Society
DYD: Down Your Drink
GDPR: General Data Protection Regulation
GHQ-28: General Health Questionnaire-28
GP: general practitioner
HE: health economics
HRA: Health Research Authority
IDMEC: independent data monitoring and ethics committee
IP: internet protocol
NHS: National Health Service
NICE: National Institute for Health and Care Excellence
PECR: Privacy and Electronic Communications Regulations
PIS: participant information sheet
PPI: patient and public involvement
QR: quick response
RAG: Relatives Advisory Group
RCT: randomized controlled trial
RD: resource directory
REACT: Relatives Education and Coping Toolkit
TAU: treatment as usual
TSC: Trial Steering Committee
